# CDP138 silencing inhibits TGF-*β*/Smad signaling to impair radioresistance and metastasis via GDF15 in lung cancer

**DOI:** 10.1038/cddis.2017.434

**Published:** 2017-09-07

**Authors:** Yanwei Lu, Jia Ma, Yan Li, Jing Huang, Sheng Zhang, Zhongyuan Yin, Jinghua Ren, Kai Huang, Gang Wu, Kunyu Yang, Shuangbing Xu

**Affiliations:** 1 Cancer Center, Union Hospital, Tongji Medical College, Huazhong University of Science and Technology, Wuhan 430022, China; 2 Clinic Center of Human Gene Research, Union Hospital, Tongji Medical College, Huazhong University of Science and Technology, Wuhan 430022, China; 3 Department of Cardiology, Union Hospital, Tongji Medical College, Huazhong University of Science and Technology, Wuhan 430022, China

## Abstract

CDP138, a CDK5 binding partner, regulates cell proliferation and migration. However, the mechanisms by which CDP138 functions in these processes remain unclear. In this study, we show that CDP138 is frequently overexpressed and that high levels of CDP138 are correlated with lymph node metastasis in lung cancer. Furthermore, we provide evidence that CDP138-depleted lung cancer cells exhibit enhanced radiosensitivity as well as reduced migration and invasion. Mechanistically, we identify GDF15, a member of the TGF-*β* superfamily, as a key downstream effector of CDP138. CDP138 silencing attenuates TGF-*β*/Smad signaling activation at least in part through the downregulation of GDF15. More importantly, the observed phenotypes caused by CDP138 knockdown are partially dependent on GDF15 inhibition. Together, our findings demonstrate that CDP138 positively modulates the TGF-*β*/Smad signaling pathway via GDF15 to promote radioresistance and metastasis, suggesting CDP138 as a potential oncogenic biomarker and a promising therapeutic target in the treatment of lung cancer.

Lung cancer is the most common malignancy and the leading cause of cancer death worldwide.^1, 2^ To date, surgery, radiochemotherapy and personalized targeted therapy remain the main treatment for this type of cancer.^3^ However, radioresistance and distant metastasis are considered critical causes of the failure to cure cancer. Therefore, intense efforts are needed to better understand the oncogenesis of lung cancer and to identify novel therapeutic targets.

Transforming growth factor (TGF)-*β* is a key player in the regulation of cellular proliferation, differentiation, motility, invasion, apoptosis and immune responses.^4, 5, 6^ Smad proteins are the intracellular effectors of TGF-*β* signals. Once phosphorylated, Smad proteins become activate and translocate to the nucleus, ultimately inducing the transcriptional activation of downstream target genes.^7, 8, 9^ To date, numerous studies have demonstrated that aberrant TGF-*β*/Smad signaling is involved in tumor metastasis, epithelial–mesenchymal transition (EMT) and DNA damage response.^10, 11, 12^ For example, Smad2 and Smad7 have been reported to participate in DNA damage response in an ataxia-telangiectasia mutated (ATM)-dependent manner. Inhibition of TGF-*β* signaling was found to enhance radiosensitivity in glioblastoma, breast cancer and lung cancer.^13, 14, 15, 16^ Moreover, the involvement of Smad2 in EMT through the increase in Snail expression has also been documented.^17^ The above findings support the notion that TGF-*β*/Smad signaling has a pivotal role in tumorigenesis.

C2 domain-containing phosphoprotein (CDP138), also known as KIAA0528, has been reported to be a substrate for Akt2, and it is involved in the regulation of GLUT4 vesicle-plasma membrane fusion in response to insulin.^18^ In contrast, another study demonstrated that CDP138 is not involved in GLUT4 translocation.^19^ Our previous study has shown that CDP138 is a CDK5- and FIBP-interacting protein, and these three molecules can form a stable complex that is involved in cell proliferation and migration.^20^ However, the clinical significance and mechanism by which CDP138 participates in these processes remain poorly understood.

In this study, we show that CDP138 protein levels are frequently upregulated and that elevated expression of CDP138 is correlated with lymph node metastasis in lung cancer. Furthermore, CDP138 silencing impairs radioresistance and metastasis in lung cancer cells. More importantly, we demonstrate for the first time that growth differentiation factor 15 (GDF15) acts as a downstream mediator of CDP138-induced TGF-*β*/Smad signaling activation and mediates the biological effects of CDP138 in lung cancer cells.

## Results

### CDP138 protein level is upregulated in lung cancer cell lines and tissues and is related to lymph node metastasis

**Figure 1:**
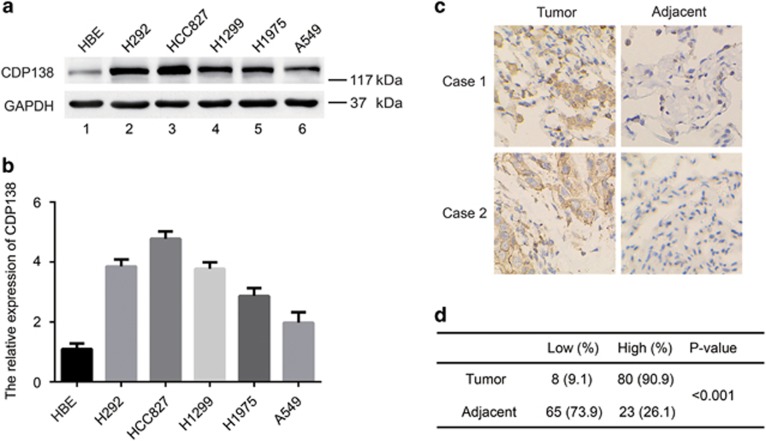
CDP138 is overexpressed in lung cancer cell lines and tissues. (**a**) Western blotting analysis of CDP138 expression in five human lung cancer cell lines and one normal human bronchial epithelial cell line. (**b**) Quantification of CDP138 protein expression in (**a**). (**c**) Representative IHC images of staining of CDP138 in tissue microarrays constructed from lung cancer and paired para-carcinoma tissues. (**d**) Summary of IHC staining for CDP138 expression in tissue microarrays. Differences between variables were assessed using the *χ*^2^ test

We previously showed that knocking down CDP138 suppresses cell growth in breast cancer,^20^ implying that CDP138 can act as an oncogenic protein. To investigate the expression of CDP138 in human lung cancer, we first analyzed CDP138 protein level in five human lung cancer cell lines (H1299, HCC827, H292, A549 and H1975) and found it to be significantly higher than that in the normal human bronchial epithelial cell line HBE (Figures 1a and b). Next, we performed immunohistochemical (IHC) analysis of CDP138 expression in a human lung cancer tissue microarray, containing 88 carcinoma tissues and paired para-carcinoma tissues. As shown in Figures 1c and d, CDP138 was primarily located in the cytoplasm and was highly expressed in lung cancer tissues. CDP138 positivity was significantly higher in lung cancer tissues (90.9%) than that in adjacent para-carcinoma tissues (26.1%) (*P*<0.001). Furthermore, there was a significant correlation between CDP138 expression and lymph node metastasis (*P*<0.05) (Table 1). However, there was no significant correlation between CDP138 expression and overall survival in lung cancer patients as determined using the Kaplan–Meier survival analysis (data not shown).

### CDP138 silencing impairs proliferation and enhances radiosensitivity in lung cancer cells

**Figure 2:**
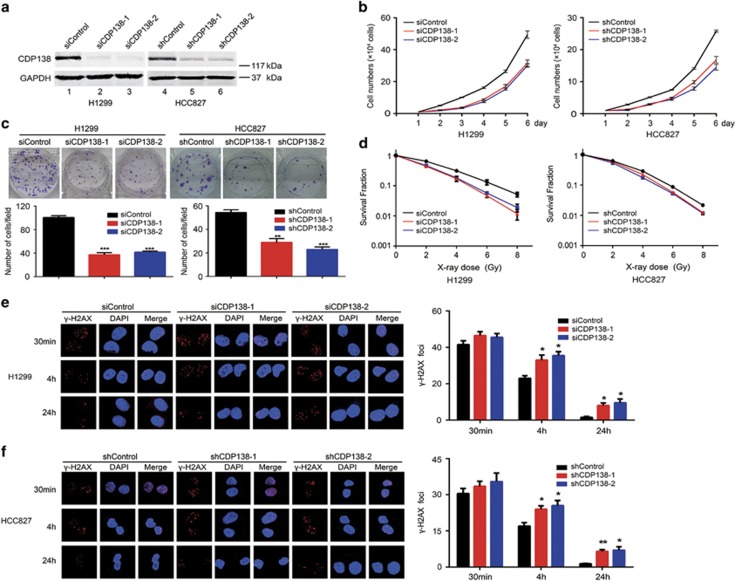
CDP138 silencing results in impaired proliferation and enhanced radiosensitivity in lung cancer cells. (**a**) Western blotting analyses revealed that CDP138 is efficiently knocked down in H1299 and HCC827 cells. (**b**) Knocking down CDP138 suppresses cell growth. H1299 and HCC827 cells were seeded, and cell numbers were evaluated every day. (**c**) Colony formation is significantly reduced in CDP138-depleted cells. **P<0.01 and ***P<0.001 compared with controls cells. (**d**) Radiation sensitivity of H1299 and HCC827 cells lacking CDP138. H1299 cells transfected with indicated siRNAs and stable HCC827 cells were irradiated at indicated doses. Percentages of surviving colonies were examined two weeks later. (**e**,**f**) Left panel: H1299 cells (**e**) or HCC827 cells (**f**) were exposed to 2 Gy radiation and harvested at indicated time points. Immunostaining was performed to determine *γ*-H2AX foci formation. Right panel: Quantification of *γ*-H2AX foci in H1299 cells (**e**) or HCC827 cells (**f**). *P<0.05, **P<0.001 compared with controls cells

Given that CDP138 is frequently overexpressed in lung cancer tissues, we speculated that it may promote tumorigenesis in lung cancer. To this end, we first downregulated the expression of CDP138 in two different lung cancer cell lines (H1299 and HCC827) using siRNAs or shRNAs. As shown in Figure 2a, CDP138 were successfully knocked down. Moreover, CDP138 silencing dramatically suppressed the proliferation of lung cancer cells compared with that of control cells (Figure 2b). The colony formation capability of the cells was also impaired when CDP138 was knocked down (Figure 2c). Therefore, CDP138 promotes the proliferation of lung cancer cells.

After determining the role of CDP138 in a physiological setting, we also wanted to know whether CDP138 is required for the same cellular processes under conditions of stress, such as irradiation (IR)-induced DNA damage. As expected, the loss of CDP138 significantly enhanced cellular sensitivity to radiation (Figure 2d), suggesting that CDP138 has an essential role in promoting radioresistance. To further confirm our hypothesis, *γ*-H2AX foci formation assay was performed since *γ*-H2AX is considered a key marker of DNA damage. As shown in Figures 2e and f, the number of *γ*-H2AX foci was significantly increased in CDP138-depleted lung cancer cells after 4 and 24 h of IR exposure. Together, these data strongly indicate that CDP138 promotes proliferation and radioresistance in lung cancer cells.

### Loss of CDP138 suppresses the migration and invasion of lung cancer cells

**Figure 3:**
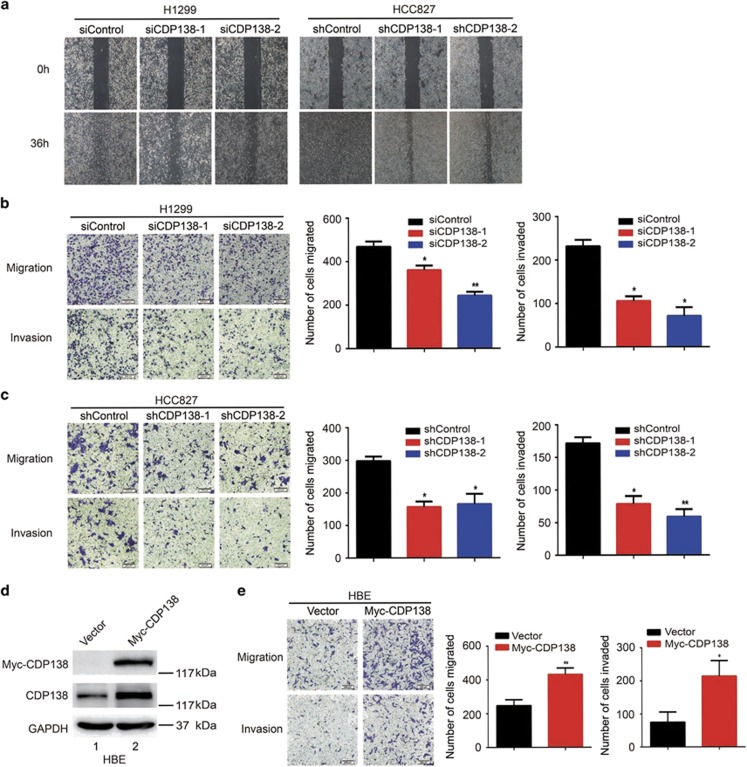
Knocking down CDP138 inhibits the migration and invasion of lung cancer cells. (**a**) Cell migration is decreased in CDP138 knockdown cells as determined using wound healing assay. (**b,c**) Left panel: Transwell assays showing that CDP138-depleted H1299 cells (**b**) or HCC827 cells (**c**) have lower migratory and invasive capacity than those of control cells. Right panel: Quantification of migration and invasion in H1299 cells (**b**) or HCC827 cells (**c**). *P<0.05 and **P<0.01 compared with controls cells. (**d**) HBE cells were transfected with the indicated plasmids. After 24 h, cells were harvested and analyzed via Western blotting with indicated antibodies. (**e**) Left panel: cell migration and invasion is measured using transwell assays. Right panel: quantification of migration and invasion in HBE cells. *P<0.05 and **P<0.01 compared with control cells

Our clinical data have clearly shown that CDP138 overexpression is correlated with lymph node metastasis (Table 1). Thus, we speculated that CDP138 may be associated with migration and invasion in lung cancer cells. First, we performed wound healing assay to determine the migratory ability of lung cancer cells. As shown in Figure 3a, CDP138 knockdown cells migrated at a slower rate than the control cells. These phenotypes were further confirmed using transwell assays. As shown in Figures 3b and c, depletion of CDP138 dramatically inhibited cellular migration and invasion. In addition, we found that the overexpression of CDP138 in normal lung epithelial (HBE) cells significantly enhanced their migration and invasion (Figures 3d and e). Thus, CDP138 enhances migration and invasion in lung cancer cells.

### CDP138 knockdown attenuates the TGF-*β*/Smad signaling pathway at least in part via the downregulation of GDF15

**Figure 4:**
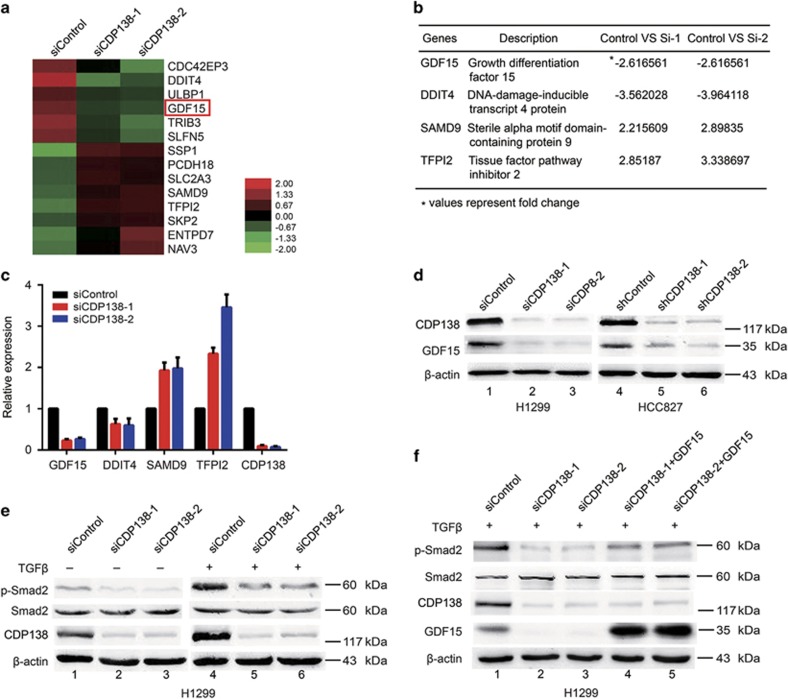
CDP138 knockdown attenuates the TGF-*β*/Smad signaling pathway at least in part via the downregulation of GDF15. (**a**) Heat map produced from mRNA microarray analysis. H1299 cells were transfected with indicated siRNAs for 48 h, and mRNA was isolated and evaluated using microarray analysis. (**b**) List of differentially expressed genes related to cell proliferation and metastasis (fold change>2). (**c**) H1299 cells were transfected with indicated siRNAs for 48 h and then harvested. Levels of indicated mRNAs were determined using quantitative real-time PCR. (**d**) GDF15 protein level was dramatically reduced in CDP138-depleted cells. (**e**) Knockdown of CDP138 decreases p-Smad2 protein expression. H1299 cells transfected with indicated siRNAs were treated with TGF-*β* (10 ng/ml) for 24 h. Cells were harvested and subjected to western blotting with indicated antibodies. (**f**) GDF15 overexpression partially rescues the reduction in p-Smad2 level. H1299 cells were transfected with indicated siRNAs for 24 h, and GDF15 expression plasmid was introduced. Cells were harvested after TGF-*β* treatment (10 ng/ml), and the lysates were subjected to western blotting with indicated antibodies

To elucidate the underlying mechanisms by which CDP138 functions in lung cancer, we performed microarray analysis to compare the genomic expression profiles of H1299 cells transfected with control or CDP138-targeting siRNAs. As shown in Figure 4a, there were 14 differentially expressed genes with more than twofold change (six were upregulated, and the rest were downregulated). Among these, we selected four candidate genes (GDF15, DDIT4, SAMD9 and TFPI2) that have been reported to involved in cell proliferation and metastasis^21, 22, 23, 24^ (Figure 4b). These candidate genes were further confirmed using quantitative real-time PCR. As shown in Figure 4c, GDF15 was the most downregulated gene in response to CDP138 depletion in lung cancer cells.

Growth differentiation factor 15 (GDF15), also known as macrophage inhibitory cytokine-1 (MIC-1), is a member of the TGF-*β* superfamily.^21^ Western blotting showed that CDP138 knockdown significantly downregulated GDF15 expression in H1299 and HCC827 cells (Figure 4d). Accumulating evidence has shown that GDF15 is involved in the regulation of the TGF-*β*/Smad signaling pathway.^25, 26^ Thus, we investigated whether CDP138 is associated with this classical signaling pathway. As shown in Figure 4e, the protein levels of p-Smad2 was reduced in the CDP138-deficient lung cancer cells, whereas the level of total Smad2 remained constant. Importantly, these effects induced by CDP138 knockdown could be partially rescued by GDF15 overexpression (Figure 4f). Therefore, CDP138 silencing attenuates the TGF-*β*/Smad signaling pathway at least in part via the downregulation of GDF15.

### Enhanced radiosensitivity and decreased migration in cells caused by CDP138 knockdown are partially dependent on GDF15 inhibition

**Figure 5:**
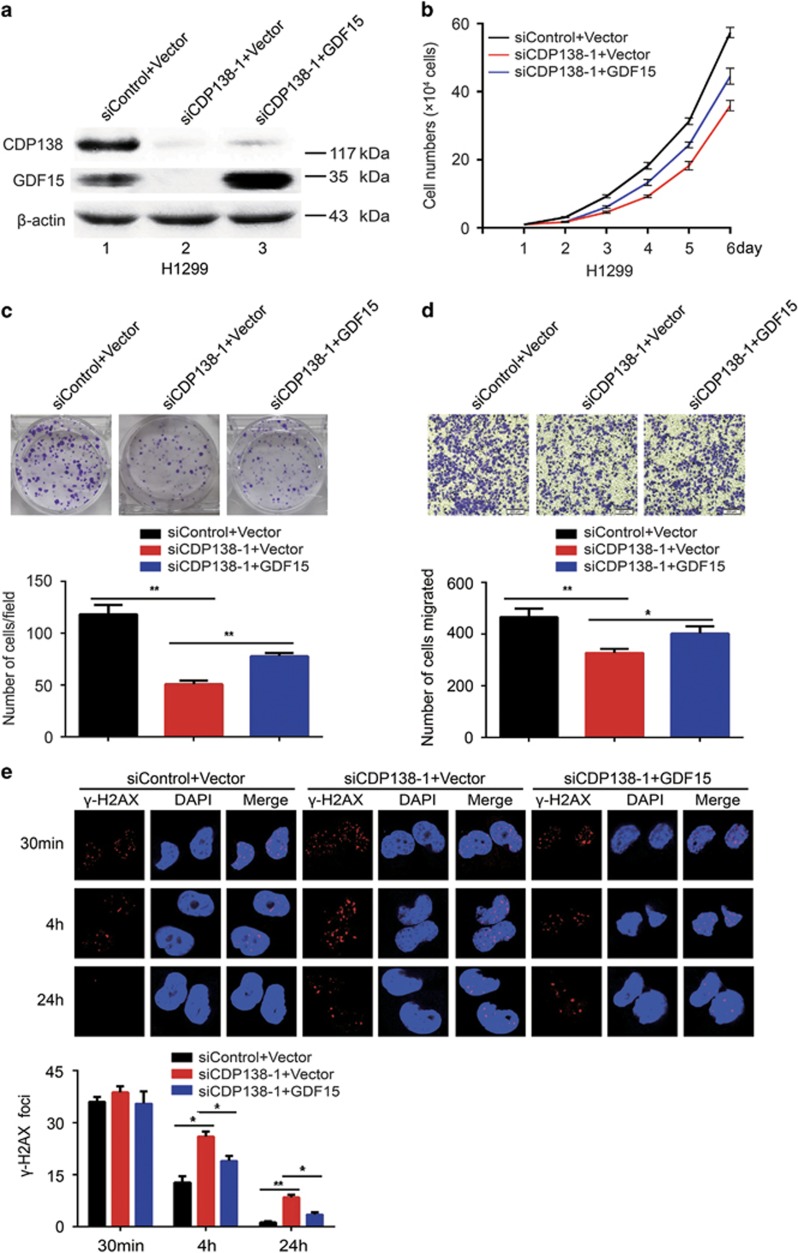
GDF15 overexpression partially rescues the impaired radioresistance and migration in lung cancer cells. (**a**) H1299 cells were transfected with the indicated siRNAs. After 24 h, the cells were then transfected with GDF15 expression plasmids. After 24 h, the cells were harvested and analyzed via western blotting. (**b**) GDF15 partially rescues the defects in cell growth. H1299 cells transfected with indicated siRNAs and plasmids were seeded, and cell numbers were evaluated every day. (**c**) Upper panel: H1299 cells were seeded and grown for two weeks. Number of colonies was counted. Lower panel: quantification of colony formation. (**d**) Upper panel: cell migration is determined using transwell assays. Lower panel: quantification of migration in H1299 cells. (**e**) Upper panel: H1299 cells transfected with indicated siRNAs and plasmids were exposed to 2 Gy radiation and harvested at the indicated time points. Immunostaining was performed to determine *γ*-H2AX foci formation. Lower panel: quantification of *γ*-H2AX foci in H1299 cells. *P<0.05 and **P<0.01. n.s. indicates no statistically significant difference (*P*>0.05)

**Figure 6:**
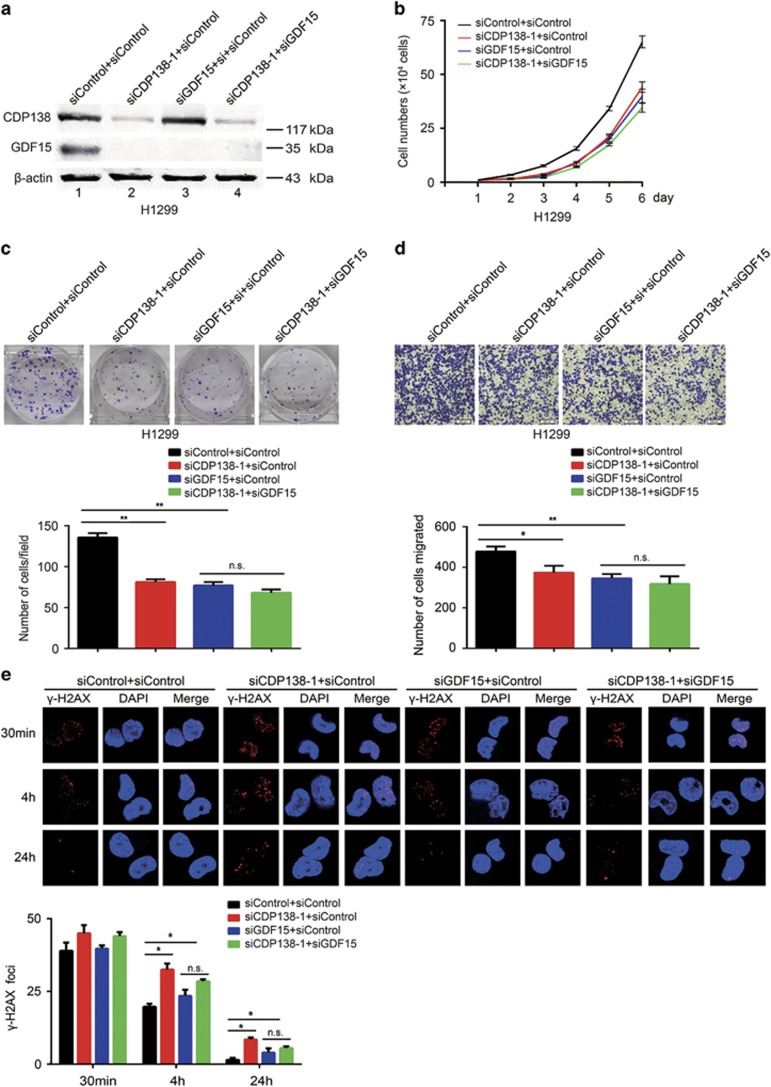
GDF15 is required for radioresistance and migration in CDP138-depleted H1299 cells. (**a**) H1299 cells were transfected with the indicated siRNAs. After 48 h, the cells were harvested and analyzed via Western blotting. (**b**) H1299 cells transfected with the indicated siRNAs were seeded at low density, and cell numbers were evaluated every day. (**c**) Upper panel: H1299 cells were transfected with indicated siRNAs and grown for two weeks. Number of colonies was counted. Lower panel: quantification of colony formation. (**d**) Upper panel: cell migration is determined using transwell assays. Lower panel: quantification of migration in H1299 cells. (**e**) Upper panel: H1299 cells transfected with indicated siRNAs were exposed to 2 Gy radiation and harvested at the indicated time points. Immunostaining was performed to examine *γ*-H2AX foci formation. Lower panel: quantification of *γ*-H2AX foci in H1299 cells. *P<0.05 and **P<0.01. n.s. indicates no statistically significant difference (*P*>0.05)

To determine whether GDF15 is indeed required for the observed phenotypes induced by CDP138 knockdown in lung cancer cells, the following rescue experiments were performed. First, we transfected exogenously expressed GDF15 to CDP138 knockdown cells (Figure 5a). As shown in Figures 5b–d, the defects in proliferation and migration induced by CDP138 knockdown in H1299 cells was partially rescued by the overexpression of GDF15. Similarly, restoring GDF15 expression rescued the IR-induced DNA damage response (*γ*-H2AX foci formation) in CDP138-depleted cells (Figure 5e). To further confirm our working hypothesis, we examined the effect of CDP138 knockdown on GDF15-depleted lung cancer cells. Western blotting showed that both CDP138 and GDF15 were efficiently depleted in H1299 cells (Figure 6a). As shown in Figures 6b–d, GDF15 knockdown significantly suppressed proliferation and migration in lung cancer cells. Meanwhile, the *γ*-H2AX foci formation was increased in the GDF15-depleted cells (Figure 6e). This is consistent with GDF15 being a major positive regulator of the TGF-*β*/Smad signaling pathway. However, CDP138 knockdown had minimal effect on the migration and radioresistance of GDF15-depleted lung cancer cells (Figures 6b–e). Therefore, the enhanced radiosensitivity and reduced migratory ability caused by CDP138 knockdown are partially dependent on GDF15 inhibition.

## Discussion

In this study, we find that CDP138 protein is overexpressed and correlated with lymph node metastasis in lung cancer tissues. Furthermore, we show that CDP138 knockdown attenuates the TGF-*β*/Smad signaling pathway via GDF15, ultimately impairing radioresistance and metastasis in lung cancer. Importantly, GDF15 is identified as a critical downstream mediator of CDP138, indicating that the CDP138/GDF15/TGF-*β* pathway is a potential therapeutic target in lung cancer.

CDP138 was first identified as an AKT2 downstream substrate required for GLUT4 translocation.^18^ Our previous study has demonstrated that CDP138 participates in cell growth and migration in breast cancer.^20^ However, little is known about the roles of CDP138 in tumorigenesis, especially in lung cancer. Our results revealed that CDP138 is overexpressed in lung cancer and associated with lymph node metastasis, strongly indicating that CDP138 may be an oncoprotein involved lung cancer metastasis. Further functional studies confirmed this notion and showed that depletion of CDP138 impaired cell proliferation both under physiological conditions and in response to DNA damage and inhibited cell migration and invasion. This finding suggests that CDP138 can contribute to radioresistance and metastasis in lung cancer.

As a member of the TGF-*β* superfamily, GDF15 has been shown to have important roles in diverse cellular processes such as proliferation, migration, inflammation, metabolism and DNA damage response.^21^ Several studies have shown that GDF15 is a radiation-induced biomarker that promotes radioresistance.^27, 28^ The role of GDF15 in promoting metastasis has also been reported.^29^ In addition, GDF15 has been found to be regulated by several critical molecules or signaling pathways. For example, the PI3K/AKT/GSK-3*β* pathway has been shown to regulate GDF15 expression at both mRNA and protein levels.^30^ The transcription factor p53 has also been reported to be required for the induction of GDF15 expression.^31^ In our study, we identified GDF15 as a key downstream mediator using microarray analysis. Our results also showed that the expression of GDF15 is regulated by CDP138 at both transcriptional and post-translational levels. Importantly, we found that CDP138 silencing attenuates the TGF-*β*/Smad signaling pathway partially via GDF15. Therefore, GDF15 likely mediates the biological consequences of CDP138 knockdown in lung cancer cells. Hence, our study identified CDP138 as a novel regulator of the TGF-*β*/Smad signaling pathway and provided new insights into the mechanisms by which CDP138 functions in the radioresistance and metastasis of lung cancer.

In conclusion, we elucidated the functions of CDP138 in lung cancer and demonstrated that CDP138 affects biological processes mainly via the GDF15-mediated TGF-*β*/Smad signaling pathway. Given that CDP138 is frequently overexpressed and causes radioresistance and metastasis in lung cancer, it may be a promising therapeutic biomarker for cancer radiotherapy, especially in lung cancers displaying elevated levels of CDP138.

## Materials and methods

### Cell cultures

Five human lung cancer cell lines (H1299, HCC827, H292, A549 and H1975) and one normal human bronchial epithelial cell line HBE were purchased from American Type Culture Collection and maintained in RPMI-1640 with 10% fetal bovine serum and 100 *μ*g/ml penicillin and streptomycin at 37 °C in 5% CO_2_. TGF-*β* was purchased from Calbiochem and stored at −20 °C.

### RNA interference

The sequences of oligonucleotides targeting mRNA are as follows: CDP138 siRNA-1, 5′-GCUAUAGAGCUGUGAUAAU-3′ CDP138 siRNA-2, 5′-GCAGCAUUCCUUCCUGCAU-3′ and GDF15 siRNA, 5′-CCAACUGCUGGCAGAAUCU-3′. H1299 cells were transfected with 100 nM siRNAs using Lipofectamine RNAiMAX reagent (Invitrogen, Camarillo, CA, USA).

### Establishment of stable lung cancer cell lines

The shRNA sequences have been previously described.^20^ HEK293T cells were transiently transfected with CDP138 shRNAs and packaging plasmids pSPAX2 and pMD2G (kindly provided by Dr Zhou Songyang, Baylor College of Medicine). At 48 h post-transfection, the lentiviral supernatants were filtered and used to infect HCC827 cells in the presence of 8 *μ*g/ml polybrene. Stable cell lines were selected with media containing 2 *μ*g/ml puromycin and confirmed by Western blotting.

### Gene expression microarrays

H1299 cells were transfected with control or CDP138-targeting siRNAs using Lipofectamine RNAiMAX for 48 h. Total RNA was isolated using Trizol reagent (Invitrogen) according to the manufacturer’s instructions. Microarray experiments were performed using Affymetrix gene chip. Genes were determined to be significantly differentially expressed with a selection threshold of false discovery rate (FDR) was<5% and fold change was >2.0. The 8 most upregulated and 6 most downregulated genes are presented as heat maps.

### Quantitative real-time PCR

This assay was performed as previously described.^32^ Briefly, total RNA was prepared using Trizol reagent. First-strand cDNA was synthesized using the qPCR RT Master Mix (Toyobo, Osaka, Japan). The relative gene expression levels were calculated using the ΔCt method (Ct of GAPDH minus the Ct of the target genes). Primer sequences are listed in Supplementary Table 1.

### Western blotting

Cell lysates were prepared using NETN buffer (20 mM Tris-HCl, pH 8.0, 100 mM NaCl, 1 mM EDTA and 0.5% Nonidet P-40), separated by SDS-PAGE and transferred to PVDF membranes. The lysates were then incubated with primary antibodies against CDP138 (Bethyl Laboratories, Montgomery, TX, USA), GDF15 (Santa Cruz Biotechnology, Santa Cruz, CA, USA), p-Smad2 (Cell Signalling Technology, Beverly, MA, USA), Smad2 (Abclone, Wuhan, China), GAPDH (Santa Cruz Biotechnology) and *β*-actin (Sigma-Aldrich, St. Louis, MO, USA) overnight at 4 °C. The samples were then incubated with secondary antibodies and visualized via enhanced chemiluminescence.

### Cell growth and colony formation assay

Cells were seeded at a density of 1.0 × 10^4^ cells/ml in 6-well plates, and cell numbers were evaluated every day. Alternatively, cells were plated in triplicate in six-well plates and irradiated at indicated doses. After being grown two weeks, the cells were stained with crystal violet, and the number of colonies (clusters of >50 cells) was calculated. The data presented represent the mean of all measured points±S.D.

### Wound healing assay

This assay was performed as previously described.^20^ Briefly, confluent cell layers were scratched with 200-*μ*l pipet tips, washed twice with PBS to remove detached cells, and then incubated in serum-free medium. Images were photographed 36 h later with a microscope.

### Cell migration and invasion assays

This assay was performed as previously described.^20^ Briefly, for migration assays, 4.0 × 10^4^ cells in 200 *μ*l of serum-free RPMI-1640 medium were added to the upper chambers of transwell plates. For invasion assays, 10.0 × 10^4^ cells in 200 *μ*l of serum-free RPMI-1640 medium were added to the upper chamber of transwell plates with Matrigel-coated membranes. Medium containing 10% FBS was added to the bottom chamber. After incubation for 24 h, the migrated and invaded cells were fixed and stained with crystal violet. The stained cells were photographed and counted.

### Immunofluorescence staining

Cells were cultured on coverslips and exposed to 2 Gy radiation. The cells were then fixed with 4% paraformaldehyde solution at various time points after IR (30 min, 4 h and 24 h) and permeabilized with 0.2% Triton X-100 for 5 min. After being blocked with 5% bovine serum albumin, the samples were incubated with anti-*γ*-H2AX antibody overnight at 4 °C. The cells were then washed and incubated with secondary antibodies for 1 h. After being counterstained with DAPI, immunostained cells were examined with a fluorescence microscope.

### IHC staining

A lung cancer tissue microarray was obtained from Shanghai Outdo Biotech (Shanghai, China), which contained 90 carcinoma tissues and paired para-carcinoma tissues. All patients were pathologically diagnosed with lung adenocarcinoma. IHC analysis was performed as previously described.^33^ Briefly, tissue sections were blocked with goat serum after antigen retrieval using citrate buffer. Then, tissue sections were incubated with anti-CDP138 antibody (Bethyl Laboratories) overnight at 4 °C. After being incubated with secondary antibody, the tissue sections were washed with PBS, reacted with 3,3-diaminobenzidine and counterstained with haematoxylin. Both the percentage of positive area and staining intensity were recorded to evaluate the expression of CDP138.

### Statistical analyses

Each experiment in our study was independently performed at least three times. Unpaired Student’s *t*-test was used for statistical comparison. Differences between variables were assessed using the *χ*^2^ test. The Kaplan–Meier method was used to construct the survival curves. *P-*value<0.05 was considered statistically significant.

## Statistical analyses

Publisher’s Note: Springer Nature remains neutral with regard to jurisdictional claims in published maps and institutional affiliations.

## Figures and Tables

**Table 1:** Correlation between the clinicopathologic variables and expression of CDP138 in human lung cancer tissues

**Features**	**CDP138 expression**		***P*-value**
	**Low**	**High**	
*Sex*			
Male	3	44	0.190
Female	5	36	
			
*Age (years)*			
≤60	6	49	0.397
>60	2	31	
			
*Tumor size*			
T1 (≤3 cm)	3	30	0.155
T2 (>3 cm, ≤7 cm)	5	44	
T3 (>7 cm)	0	6	
			
*Stage*			
I–II	5	63	0.912
III–IV	3	17	

*T*			
1	2	15	0.591
2	4	45	
3	1	16	
4	1	4	
			
*N*			
0	5	41	**0.043**
1	0	19	
2	2	14	
3	1	6	
			
*M*			
0	8	79	0.151
1	0	1	

## References

[bib1] Torre LA, Bray F, Siegel RL, Ferlay J, Lortet-Tieulent J, Jemal A. Global cancer statistics, 2012. CA Cancer J Clin 2015; 65: 87–108. 25651787 10.3322/caac.21262

[bib2] Chen W, Zheng R, Baade PD, Zhang S, Zeng H, Bray F et al. Cancer statistics in China, 2015. CA Cancer J Clin 2016; 66: 115–132. 26808342 10.3322/caac.21338

[bib3] Begg AC, Stewart FA, Vens C. Strategies to improve radiotherapy with targeted drugs. Nat Rev Cancer 2011; 11: 239–253. 21430696 10.1038/nrc3007

[bib4] Massague J. TGFβ in cancer. Cell 2008; 134: 215–230. 18662538 10.1016/j.cell.2008.07.001 PMC3512574

[bib5] Wu MY, Hill CS. Tgfβ superfamily signaling in embryonic development and homeostasis. Dev Cell 2009; 16: 329–343. 19289080 10.1016/j.devcel.2009.02.012

[bib6] Massague J. TGFβ signalling in context. Nat Rev Mol Cell Biol 2012; 13: 616–630. 22992590 10.1038/nrm3434 PMC4027049

[bib7] Massagué J, Blain SW, Lo RS. TGFβ signaling in growth control, cancer, and heritable disorders. Cell 2000; 103: 295–309. 11057902 10.1016/s0092-8674(00)00121-5

[bib8] Ikushima H, Miyazono K. TGFbeta signalling: a complex web in cancer progression. Nat Rev Cancer 2010; 10: 415–424. 20495575 10.1038/nrc2853

[bib9] Weiss A, Attisano L. The TGFβ superfamily signaling pathway. Wiley Interdiscip Rev Dev Biol 2013; 2: 47–63. 23799630 10.1002/wdev.86

[bib10] Barcellos-Hoff MH, Cucinotta FA. New tricks for an old fox: impact of TGFβ on the DNA damage response and genomic stability. Sci Signal 2014; 7: ref5. 10.1126/scisignal.2005474 25185158

[bib11] Derynck R, Saeteurn KY, Muthusamy BP. Signaling pathway cooperation in TGF-β-induced epithelial-mesenchymal. Curr Opin Cell Biol 2014; 31: 56–66. 25240174 10.1016/j.ceb.2014.09.001 PMC4657734

[bib12] Centurione L, Aiello FB. DNA repair and cytokines: TGF-beta, IL-6, and thrombopoietin as different biomarkers of radioresistance. Front Oncol 2016; 6: 175. 27500125 10.3389/fonc.2016.00175 PMC4956642

[bib13] Bouquet F, Pal A, Pilones KA, Demaria S, Hann B, Akhurst RJ et al. TGFβ1 inhibition increases the radiosensitivity of breast cancer cells *in vitro* and promotes tumor control by radiation *in vivo*. Clin Cancer Res 2011; 17: 6754–6765. 22028490 10.1158/1078-0432.CCR-11-0544 PMC3724539

[bib14] Zhang M, Kleber S, Rohrich M, Timke C, Han N, Tuettenberg J et al. Blockade of TGF-beta signaling by the TGFbetaR-I kinase inhibitor LY2109761 enhances radiation response and prolongs survival in glioblastoma. Cancer Res 2011; 71: 7155–7167. 22006998 10.1158/0008-5472.CAN-11-1212

[bib15] Hardee ME, Marciscano AE, Medina-Ramirez CM, Zagzag D, Narayana A, Lonning SM et al. Resistance of glioblastoma-initiating cells to radiation mediated by the tumor microenvironment can be abolished by inhibiting transforming growth factor-beta. Cancer Res 2012; 72: 4119–4129. 22693253 10.1158/0008-5472.CAN-12-0546 PMC3538149

[bib16] Du S, Bouquet S, Lo CH, Pellicciotta I, Bolourchi S, Parry R et al. Attenuation of the DNA damage response by transforming growth factor-beta inhibitors enhances radiation sensitivity of non-small-cell lung cancer cells *in vitro* and *in vivo*. Int J Radiat Oncol Biol Phys 2015; 91: 91–99. 25835621 10.1016/j.ijrobp.2014.09.026

[bib17] Hoot KE, Lighthall J, Han G, Lu SL, Li A, Ju W et al. Keratinocyte-specific Smad2 ablation results in increased epithelial-mesenchymal transition during skin cancer formation and progression. J Clin Invest 2008; 118: 2722–2732. 18618014 10.1172/JCI33713 PMC2447925

[bib18] Xie X, Gong Z, Mansuy-Aubert V, Zhou QL, Tatulian SA, Sehrt D et al. C2 domain-containing phosphoprotein CDP138 regulates GLUT4 insertion into the plasma membrane. Cell Metab 2011; 14: 378–389. 21907143 10.1016/j.cmet.2011.06.015 PMC3172579

[bib19] Sadacca LA, Bruno J, Wen J, Xiong W, McGraw TE. Specialized sorting of GLUT4 and its recruitment to the cell surface are independently regulated by distinct Rabs. Mol Bio Cell 2013; 24: 2544–2557. 23804653 10.1091/mbc.E13-02-0103 PMC3744946

[bib20] Xu S, Li X, Gong Z, Wang W, Li Y, Nair BC et al. Proteomic analysis of the human cyclin-dependent kinase family reveals a novel CDK5 complex involved in cell growth and migration. Mol Cell Proteomics 2014; 13: 2986–3000. 25096995 10.1074/mcp.M113.036699 PMC4223486

[bib21] Unsicker K, Spittau B, Krieglstein K. The multiple facets of the TGF-beta family cytokine growth/differentiation factor-15/macrophage inhibitory cytokine-1. Cytokine Growth Factor Rev 2013; 24: 373–384. 23787157 10.1016/j.cytogfr.2013.05.003

[bib22] Chang B, Liu G, Yang G, Mercado-Uribe I, Huang M, Liu J. REDD1 is required for RAS-mediated transformation of human ovarian epithelial cells. Cell Cycle 2009; 8: 780–786. 19221489 10.4161/cc.8.5.7887

[bib23] Ma Q, Yu T, Ren YY, Gong T, Zhong DS. Overexpression of SAMD9 suppresses tumorigenesis and progression during non-small cell lung cancer. Biochem Biophys Res Commun 2014; 454: 157–161. 25450373 10.1016/j.bbrc.2014.10.054

[bib24] Liu Z, Zhang J, Gao Y, Pei L, Zhou J, Gu L et al. Large-scale characterization of DNA methylation changes in human gastric carcinomas with and without metastasis. Clin Cancer Res 2014; 20: 4598–4612. 25009298 10.1158/1078-0432.CCR-13-3380 PMC4309661

[bib25] Unal B, Alan S, Bassorgun CI, Karakas AA, Elpek GO, Ciftcioglu MA. The divergent roles of growth differentiation factor-15 (GDF-15) in benign and malignant skin pathologies. Arch Dermatol Res 2015; 307: 551–577. 25690161 10.1007/s00403-015-1546-2

[bib26] Min KW, Liggett JL, Silva G, Wu WW, Wang R, Shen RF et al. NAG-1/GDF15 accumulates in the nucleus and modulates transcriptional regulation of the Smad pathway. Oncogene 2016; 35: 377–388. 25893289 10.1038/onc.2015.95 PMC4613816

[bib27] Sandor N, Schilling-Toth B, Kis E, Benedek A, Lumniczky K, Safrany G et al. Growth Differentiation Factor-15 (GDF-15) is a potential marker of radiation response and radiation sensitivity. Mutat Res Genet Toxicol Environ Mutagen 2015; 793: 142–149. 26520384 10.1016/j.mrgentox.2015.06.009

[bib28] Schiegnitz E, Kammerer PW, Rode K, Schorn T, Brieger J, Al-Nawas B. Growth differentiation factor 15 as a radiation-induced marker in oral carcinoma increasing radiation resistance. J Oral Pathol Med 2016; 45: 63–69. 25880686 10.1111/jop.12323

[bib29] Xu Q, Xu HX, Li JP, Wang S, Fu Z, Jia J et al. Growth differentiation factor 15 induces growth and metastasis of human liver cancer stem-like cells via AKT/GSK-3β/β-catenin signaling. Oncotarget 2017; 8: 16972–1687. 28199981 10.18632/oncotarget.15216 PMC5370015

[bib30] Yamaguchi K, Lee SH, Eling TE, Baek SJ. Identification of nonsteroidal anti-inflammatory drug-activated gene (NAG-1) as a novel downstream target of phosphatidylinositol 3-kinase/AKT/GSK-3beta pathway. J Biol Chem 2004; 279: 49617–49623. 15377673 10.1074/jbc.M408796200

[bib31] Cheng JC, Chang HM, Leung PC. Wild-type p53 attenuates cancer cell motility by inducing growth differentiation factor-15 expression. Endocrinology 2011; 152: 2987–2995. 21586550 10.1210/en.2011-0059

[bib32] Xu S, Wu Y, Chen Q, Cao J, Hu K, Tang J et al. hSSB1 regulates both the stability and the transcriptional activity of p53. Cell Res 2013; 23: 423–435. 23184057 10.1038/cr.2012.162 PMC3587703

[bib33] Xu S, Feng Z, Zhang M, Wu Y, Sang Y, Xu H et al. hSSB1 binds and protects p21 from ubiquitin-mediated degradation and positively correlates with p21 in human hepatocellular carcinomas. Oncogene 2011; 30: 2219–2229. 21242961 10.1038/onc.2010.596

