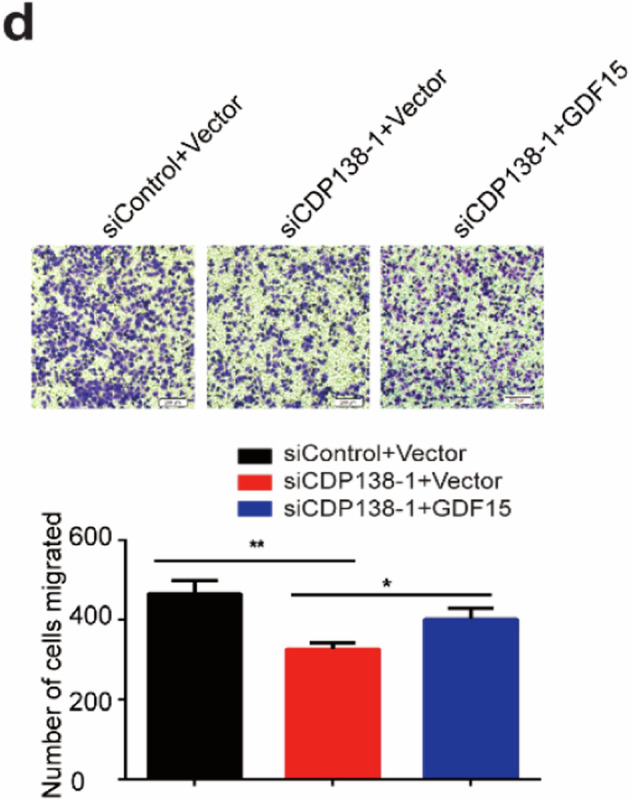# Correction: CDP138 silencing inhibits TGF-*β*/Smad signaling to impair radioresistance and metastasis via GDF15 in lung cancer

**DOI:** 10.1038/s41419-024-06823-2

**Published:** 2024-07-31

**Authors:** Yanwei Lu, Jia Ma, Yan Li, Jing Huang, Sheng Zhang, Zhongyuan Yin, Jinghua Ren, Kai Huang, Gang Wu, Kunyu Yang, Shuangbing Xu

**Affiliations:** 1 Cancer Center, Union Hospital, Tongji Medical College, Huazhong University of Science and Technology, Wuhan, 430022 China; 2 Clinic Center of Human Gene Research, Union Hospital, Tongji Medical College, Huazhong University of Science and Technology, Wuhan, 430022 China; 3 Department of Cardiology, Union Hospital, Tongji Medical College, Huazhong University of Science and Technology, Wuhan, 430022 China

Correction to: *Cell Death & Disease* 10.1038/cddis.2017.434, published online 07 September 2017

The original version of this article contained some errors. In Fig. 2c, the colony formation image of the H1299 group was inadvertently duplicated in Fig. 6c. During the figure layout, the H1299 migration images of the right panels (siCDP138-2 and siCDP138-1+GDF15) in Figs. 3b and 5d were mistakenly used due to the similarity in naming. The correct figures are provided below. Our corrections do not affect the conclusions of this article. The authors apologize for the mistakes and any inconvenience caused.

Figure 2c
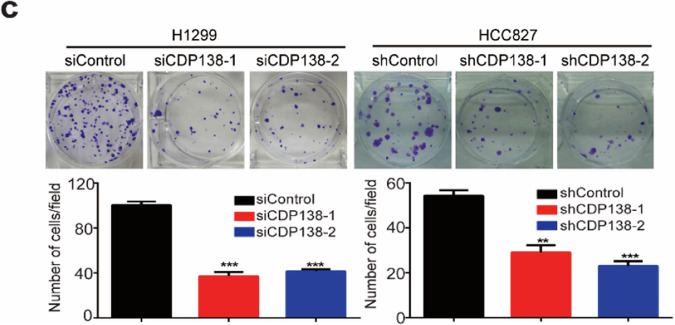


Figure 3b
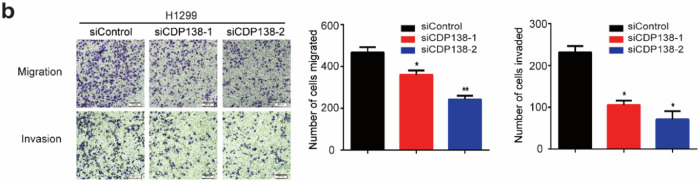


Figure 5d