# Supplementary material for: CDP138 silencing inhibits TGF-β/Smad signaling to impair radioresistance and metastasis via GDF15 in lung cancer
Source: Cell Death Dis. 2017 Sep 7;8(9):e3036–. doi: 10.1038/cddis.2017.434 (PMC5636979; doi:10.1038/cddis.2017.434)
Supplement: Supplementary Table S1 [file cddis2017434x1.doc]

**Supplementary TableS1** Sequences of primers used for Real-time quantitative PCR

| Genes | Sequences (5'--3') |
| --- | --- |
| GDF15 | F:GGTGCTCATTCAAAAGACCGA  R:CATTCCACAGGGCAGGACA |
| DDIT4 | F:CCGACAGTGCCCTCCAAG  R:TTAGGTGGCTGCCTCAGTTTT |
| SAMD9 | F:GTGGCCTTTTGTGATCTCCT  R:CTTATGACTTTCTAACCACTGA |
| TFPI2 | F:TGGTTAGCAGGGAGGATTGC  R:TCCGGATTCTACTGGCAAAG |
| CDP138 | F:TTACAGTCACGGCAGTCGCA  R:GCTGGTGGAAAAGGATGAGAA |
| GAPDH | F: GAGTCAACGGATTTGGTCGT  R: GACAAGCTTCCCGTTCTCAG |

F, forward primer; R, reverse primer.
